# CCND1 as a Predictive Biomarker of Neoadjuvant Chemotherapy in Patients with Locally Advanced Head and Neck Squamous Cell Carcinoma

**DOI:** 10.1371/journal.pone.0026399

**Published:** 2011-10-31

**Authors:** Zhien Feng, Wei Guo, Chenping Zhang, Qin Xu, Ping Zhang, Jian Sun, Hanguang Zhu, Zhonghe Wang, Jiang Li, Lizhen Wang, Bingshun Wang, Guoxin Ren, Tong Ji, Wenyong Tu, Xihu Yang, Weiliu Qiu, Li Mao, Zhiyuan Zhang, Wantao Chen

**Affiliations:** 1 Department of Oral and Maxillofacial Surgery, Shanghai Jiao Tong University School of Medicine, Ninth People's Hospital, Shanghai, China; 2 Shanghai Key Laboratory of Stomatology, Shanghai, China; 3 Department of Oral Pathology, Shanghai Jiao Tong University School of Medicine, Ninth People's Hospital, Shanghai, China; 4 Department of Biostatistics, Institute of Medical Science, Shanghai Jiao Tong University School of Medicine, Shanghai, China; 5 Department of Oncology and Diagnostic Sciences, University of Maryland Dental School, Baltimore, Maryland, United States of America; Virginia Commonwealth University, United States of America

## Abstract

**Background:**

Cyclin D1 (CCND1) has been associated with chemotherapy resistance and poor prognosis. In this study, we tested the hypothesis that CCND1 expression determines response and clinical outcomes in locally advanced head and neck squamous cell carcinoma (HNSCC) patients treated with neoadjuvant chemotherapy followed by surgery and radiotherapy.

**Methodology and Findings:**

224 patients with HNSCC were treated with either cisplatin-based chemotherapy followed by surgery and radiotherapy (neoadjuvant group, n = 100) or surgery and radiotherapy (non-neoadjuvant group, n = 124). CCND1 expression was assessed by immunohistochemistry. CCND1 levels were analyzed with chemotherapy response, disease-free survival (DFS) and overall survival (OS). There was no significant difference between the neoadjuvant group and non-neoadjuvant group in DFS and OS (p = 0.929 and p = 0.760) when patients treated with the indiscriminate administration of cisplatin-based chemotherapy. However, in the neoadjuvant group, patients whose tumors showed a low CCND1 expression more likely respond to chemotherapy (p<0.001) and had a significantly better OS and DFS than those whose tumors showed a high CCND1 expression (73% vs 8%, p<0.001; 63% vs 6%, p<0.001). Importantly, patients with a low CCND1 expression in neoadjuvant group received more survival benefits than those in non-neoadjuvant group (p = 0.016), however patients with a high CCND1 expression and treated with neoadjuvant chemotherapy had a significantly poor OS compared to those treated with surgery and radiotherapy (p = 0.032). A multivariate survival analysis also showed CCND1 expression was an independent predictive factor (p<0.001).

**Conclusions:**

This study suggests that some but not all patients with HNSCC may benefit from neoadjuvant chemotherapy with cisplatin-based regimen and CCND1 expression may serve as a predictive biomarker in selecting patients undergo less than two cycles of neoadjuvant chemotherapy.

## Introduction

Worldwide, approximately 635,000 new cases of head and neck cancer are diagnosed annually and more than 12 percents of these cases distributed in China. Unfortunately, 3 quarters of Chinese patients are already in advanced stage when diagnosed, and above 76,000 patients have been dead each year [1]. Although treatment has greatly improved in the last three decades due to advances in combined treatment, long-survival in patients with advanced head and neck squamous cell carcinoma (HNSCC), which accounts for over 80–90% of malignant tumors, is poor.

Neoadjuvant chemotherapy, which is the use of systemic chemotherapy before definitive surgery and/or radiotherapy, has been an attractive approach in the management of HNSCC for the last 25 years [2]. The benefits of chemotherapy for patients with advanced HNSCC, as demonstrated by many clinical studies, include a reduction in the distant metastasis, improved long-survival, and the preservation of organ function [3], [4], [5], [6]. Unfortunately, some studies have failed to demonstrate any significant improvement in long-survival after neoadjuvant chemotherapy [5], [7], [8]. Recently, Glynne-Jones et al. stated that there was no benefit in overall survival from cisplatin-based chemotherapy before radiotherapy and considered that neoadjuvant chemotherapy might be the sole effective preoperative management strategy in HNSCC [9], [10]. However, some studies have also shown that patients whose disease responded to neoadjuvant chemotherapy had a better survival rate in comparison those who did not receive chemotherapy or who received non-effective chemotherapy [11], [12], [13], [14], [15]. Furthermore, it has been shown that neoadjuvant chemotherapy can increase the effectiveness of radiotherapy [16]. Thus, neoadjuvant chemotherapy has become an area of intense study in HNSCC management; however, the original, empiric-based treatment strategies that have been historically used have resulted in many patients with chemotherapy-resistant disease, such that these patients frequently received multiple cycles of toxic therapy without success before the apparent lack of efficacy was identified [17].

It is believed that the extreme biological heterogeneity that defines the chemotherapy-resistant phenotype and prognosis differs among patients and generally involves many factors. Accumulating evidence indicates that a high expression of cyclin D1 (CCND1), which is a key regulator of the G1 phase of the cell cycle, is associated with chemotherapy resistance and a poor prognosis in some solid malignant tumors [18], [19], [20]. Our previous studies have also found that a high expression of CCND1 in HNSCC was closely associated with cisplatin resistance *in vitro* and *in vivo* [21], [22]. These results have led us to hypothesize that CCND1 could be an important target for chemotherapy response and monitoring prognosis in patients with locally advanced HNSCC.

In the present study, we developed a predictive assay that is capable of selecting patients who would receive the largest possible benefit from cisplatin-based chemotherapy before surgery and post-operative radiotherapy. As a proof of principle, we investigated the direct link between CCND1 protein expression and the treatment efficacy in patients with locally advanced HNSCC. Ultimately, our main goal was to obtain preliminary data on CCND1 expression, and to evaluate its potential as an independent molecular predictor for developing personalized treatment plans for patients with HNSCC.

## Methods

### Patient samples

All of the patients gave written informed consent in accordance with institutional guidelines. During January 1999 to March 2005, all patients with HNSCC, being pathologically diagnosed squamous cell carcinoma, who were treated at the Department of Oral and Maxillofacial Surgery, were screened for the study. Patients inclusion criteria included: (1) a primary and moderately advanced tumor (clinical stage III/IVa; UICC/AJCC. 7ed., 2010); (2) complete medical information and follow-up data; (3) a Karnofsky performance score of at least 60; (4) a WBC count of greater than 4000/mm^3^, a platelet count ≥100,000/mm^3^, a normal serum calcium level, and a creatine clearance ≥55 ml/min; (5) well-compensated or no pulmonary disease as documented by pulmonary function tests (if receiving pingyangmycin); (6) no previous treatment. The identifier data were terminally coded in order to maintain patient anonymity.

### Treatment protocols

#### Neoadjuvant chemotherapy

Patients in neoadjuvant group initially underwent cisplatin-based intravenous chemotherapy. For the cisplatin-based regimen, patients received cisplatin (platinum-containing; cell cycle specific agent) at 80 mg/m^2^ on day 1, teniposide (derivative of podophyllotoxin) at 60 mg/m^2^ on days 2–4 or vindesine (vinca vlkaloids; mitotic inhibitor) at 1.6 mg/m^2^ on day 2, pingyangmycin (antibiotic cancer agent) at 6 mg/m^2^ on day 3–12 of a 21-day cycle. The chemotherapy regimen consisted of one to two cycles.

#### Surgery

Patients underwent radical tumor resection within two to three weeks of completing chemotherapy or within one week after enrolling in non-neoadjuvant group. The surgical procedure was selected by surgeons according to tumor site and local practice. Standardized surgery, including radical tumor resection, neck lymph node dissection and the reconstruction of tissue defects (as necessary), was performed.

#### Radiotherapy

After surgical resection, all patients received post-operative radiotherapy. Patients underwent radiotherapy within two to six weeks of completing surgery. The conventional radiotherapy regimen of five fractions per week from Monday to Friday with 200 cGy per day was administered. Total dose: primary tumor area and neck of positive nodes >6000 cGy, neck of negative nodes >5000 cGy, and positive tumor margins >6500 cGy.

### Clinical outcome assessment of neoadjuvant chemotherapy

The clinical responses to chemotherapy were evaluated no less than two weeks after patients completed chemotherapy according to response evaluation criteria in solid tumors (RECIST) [23].

Patients were evaluated for treatment-related toxicity at a minimum of every seven days according to the National Cancer Institute Common Toxicity Criteria, version 2.0. The grade of toxicity per patient was recorded.

### Immunohistochemistry

Immunohistochemistry (IHC) was performed using a rabbit monoclonal antibody against the CCND1 protein [Epitomics, Inc., United States; Clone-EPR2241 (IHC)-32] and a mouse monoclonal antibody against human pan-cytokeratin (P-CK) protein (Santa Cruz Biotechnology, Inc; sc-71838) on 3-µm slides using 224 paraffin sections via the standard SP method.

The expression level was assessed by manual counting that was aided by analysis via Image-pro Plus 6.0 (IPP 6.0). The measurement parameters included the area sum, density mean, and integrated optical density (IOD). To rule out the nonspecific stain of CCND1, the P-CK stain was employed in controversial section (such as suspicious non cancerous cell stain), and then the function of irregular automated optical inspection (irregular AOI) was applied by IPP 6.0 software to score. CCND1 expression was determined by counting 1,000 cells in 10 large graticules visible in the microscope. All images which were analyzed using IPP 6.0 were verified by two pathologists who were blinded to the results of the previous assessments and the two groups. When disagreement existed, a consensus was reached by discussion.

### Statistical analysis

The characteristics of patients were expressed as percentages or means. The baseline data of two groups were compared using non parametric tests, except that age was compared using independent sample *t* test. The association between CCND1 protein expression and chemotherapy response and prognosis was evaluated via Fisher's exact test. For survival analysis, automated IOD scores were converted into binomial variables of high versus low expression around the median. The OS was calculated as the period from the first day after treatment until death from any cause or until the date of the last follow-up, at which point the data were censored. The DFS was defined as the time from the first day after treatment to death from any cause or from disease progression. Survival curves were calculated using the Kaplan-Meier method. The log-rank test was used to compare the DFS and OS hazards in the two groups and the survival of CCND1 expression subgroups (high/low expression) between the two groups. Cox proportional hazard models were utilized for univariate and multivariate analyses of molecular biomarkers and other baseline factors with OS. All calculations and analyses were performed using the SAS 9.2 Statistical Package for Windows and were two-tailed where appropriate.

## Results

### Patient characteristics

All 100 eligible patients who underwent cisplatin-based neoadjuvant chemotherapy + surgery + postoperative radiotherapy were enrolled as neoadjuvant group in this study, whereas another 124 successive patients who directly underwent surgery and postoperative radiotherapy were enrolled as non-neoadjuvant group. Of the 100 patients who underwent neoadjuvant chemotherapy, 83 patients underwent a PTP regimen and 17 patients underwent a PVP regimen. The remaining 124 patients directly underwent surgery and postoperative radiotherapy. In these patients' data, the cutoff date of following-up was March 1, 2010 for survivors. The median follow-up for surviving patients was 107 months [interquartile range (IQR), 75 to 120] for neoadjuvant group and 102 months [interquartile range (IQR), 78 to 110] for non-neoadjuvant group. The patients' eligibilities were well balanced between the two groups (listed in Table 1).

**Table 1:** Baseline demographics for the 224 patients who participated in the study.

	neoadjuvant		non-neoadjuvant		
	group(n = 100)		group(n = 124)		
Variable	No	%	No	%	*P*
**Age, yrs: mean ± SD**	55.7±13.2		58.4±13.0		0.125
**Gender**					
Male	74	74.0	89	71.8	0.710
Female	26	26.0	35	28.2	
**Site**					
Tongue	38	38.0	48	38.7	0.547
Gingiva	16	16.0	25	20.2	
Buccal mucosa	19	19.0	24	19.4	
Floor of the mouth	10	10.0	10	8.1	
Oropharynx	10	10.0	11	8.9	
Hard palate	7	7.0	4	3.2	
Nasal sinuses	0	0.0	2	1.6	
**Clinical stage**					
III	37	37.0	47	37.9	0.890
IVa	63	63.0	77	62.1	
**Pathologic grade**					
I	73	73.0	96	77.4	0.423
II	23	23.0	25	20.2	
III	4	4.0	3	2.4	
**Smoking history**					
Smoker	47	47.0	56	45.2	0.863
Nonsmoker	48	48.0	60	48.4	
Missing	5	5.0	8	6.4	
**Alcohol history**					
Drinker	32	32.0	35	28.3	0.587
Nondrinker	63	63.0	81	65.3	
Missing	5	5.0	8	6.4	

Abbreviations: SD: standard deviation; neoadjuvant group: cisplatin-based neoadjuvant chemotherapy followed by surgery and radiotherapy group. Non-neoadjuvant group: surgery followed by radiotherapy group.

### Surgical specimen characteristics

The characteristics of the surgical specimens are detailed in Table 2. Exactly 116 (52%) of 224 patients had nodal involvement, with bilateral metastases or lower cervical metastases in 25 of the 116 patients. Tumor margins were histologically analyzed in 210 of 224 patients. Fifteen (7%) of the 210 margins were considered to be positive. Other histological signs of severity (vascular emboli, perineural invasion, diffuse infiltration) were present in 124 (55%) of 224 patients. As shown in Tables 1 and 2, patients were well matched between the two groups.

**Table 2:** Characteristics of surgical specimens.

	neoadjuvant		non-neoadjuvant		
	group		group		
	(n = 100)		(n = 124)		
Characteristic	No	%	No	%	*P*
**Noda histology, N+**					
No. of patients with positive nodes	52	52.0	64	51.6	0.744
1	18	18.0	21	16.9	
≥2 except bilateral or lower cervical metastases	19	19.0	33	26.6	
≥2 with bilateral or lower cervical metastases*	15	15.0	10	8.1	
**Surgical margin**					
Missing	7	7.0	7	5.6	0.729
Positive	6	6.0	9	7.3	
Negative	87	87.0	108	87.1	
**Histologic signs of severity (vascular emboli,**					
**perineural invasion, diffuse infiltration)**					
Missing	3	3.0	5	4.0	0.850
None	42	42.0	50	40.3	
Presence	55	55.0	69	55.6	

Note: neoadjuvant group: cisplatin-based neoadjuvant chemotherapy followed by surgery and radiotherapy. Non-neoadjuvant group: surgery followed by radiotherapy. Lower cervical metastases: cervical metastases below the plane of cricoid cartilage inferior margin.

### Treatment outcome

In neoadjuvant group, 88 (88%) out of 100 patients received one cycle of chemotherapy, whereas 12 (12%) patients received two cycles of chemotherapy. Among the patients, 9 (9%) had complete response (CR), 54 (54%) had partial response (PR), with the overall response rate of 63%. Twenty-five (25%) of the patients had stable disease (SD) and 12 (12%) patients had progression disease (PD). Ten (12 times) out of the 100 patients showed toxicity grade 3. The most relevant reasons of toxicity in the cisplatin-based regimens were neutropenia, vomiting and hepatotoxicity.

Radical surgery was performed for all the patients in both groups. Neck lymph node dissection was performed in 210 (94%) of the 224 patients, including unilateral regional neck dissection (n = 105), unilateral functional/radical neck dissection (FND/RND) (n = 55), bilateral regional neck dissection (n = 7), one side regional with the other side radical neck dissection (n = 30) and bilateral radical neck dissection (n = 13).

After surgery, all the patients underwent post-surgical radiotherapy. However, the radiation doses could not be determined in six of the 224 patients due to missing of clinical data. Eighteen patients voluntarily stopped radiotherapy and additional seven patients underwent dose modification due to intolerable side effects.

### CCND1 protein expression

**Figure 1:**
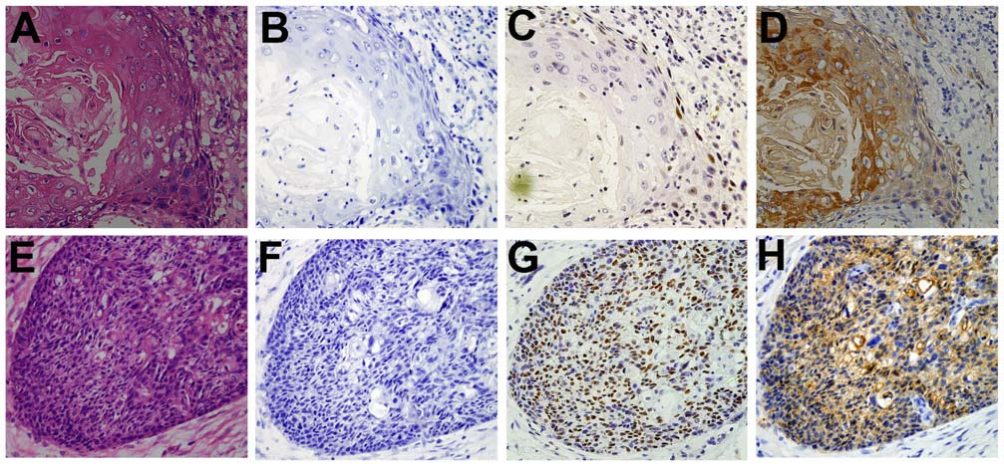
Two typical cases with low and high cyclin D1 expression were tested by cytokeratin stain. Figs. A–D: The low cyclin D1 expression case. A. HE stain (400cyclin D1 expression were tested by cytok D1 stain (400 case. P-CK stain (40000 cases. E–H: The high cyclin D1 expression case. E. HE stain (400cyclin D1 expression were tested by cytok D1 stain (400×); H. P-CK stain (40000×).

CCND1 protein expression was assessed in 224 cases by IHC; seven cases were excluded from final evaluation due to the lack of tumor cells in the tissue sections. The CCND1 protein levels in the tumors of the remaining 217 patients (100 in NG and 117 in SG) were analyzed. Thirty-eight of the 217 samples were re-evaluated by P-CK stain and the consensus scores of CCND1 expression were determined (shown in Figure 1). The median IOD of 217 patients was 31388.46, which was used as the cutoff value to determine high or low CCND1 expression. As determined by the chi-square test, there was no association between CCND1 expression and any baseline demographics.

### Neoadjuvant chemotherapy did not improve the survival of unselected patients with HNSCC

**Figure 2:**
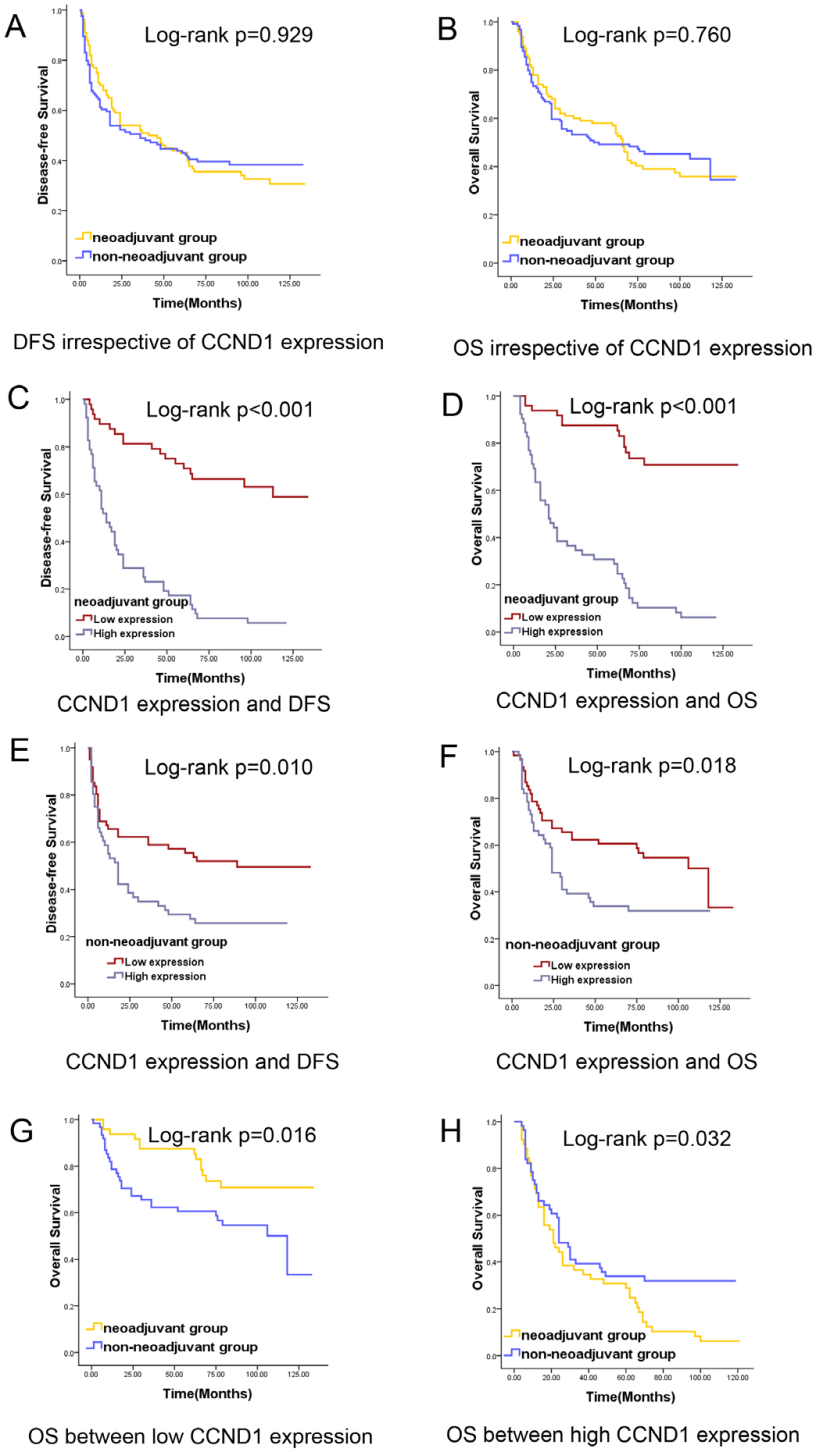
Kaplan-Meier survival curves for different treatment protocols and biomarker as well as the impact of treatment procedure according to CCND1 expression.

During follow-up period, 130 (58%) of the 224 patients had died (neoadjuvant group: 61 cases and non-neoadjuvant group: 69 cases). Eight patients died as a result of causes unrelated to cancer, including two in neoadjuvant group and six in non-neoadjuvant group. The DFS rates in neoadjuvant group and non-neoadjuvant group were 33% and 40%, and that the OS rates were 39% and 44%, respectively. Compared to conventional treatment (non-neoadjuvant group), it appears that the addition of chemotherapy did not improve the long-term survival of the patients in the whole neoadjuvant group. There was no significant difference by Kaplan-Meier analysis in DFS (p = 0.929, Fig. 2A) and OS (p = 0.760, Fig. 2B) in the two groups.

### CCND1 protein expression is significantly correlated with chemotherapy responses and clinical outcomes

The protein expression of CCND1 exhibited a significant correlation with chemotherapy response (p<0.001). A low CCND1 protein expression was closely correlated with chemotherapy response, as 41 (85%) of 48 patients with low CCND1 expressions showed clinical responses; however, a high CCND1 expression might forecast chemotherapy failure, as 30 (58%) of 52 patients with high CCND1 expression showed no clinical response (Table 3).

**Table 3:** CCND1 protein expression and clinical response in patients with HNSCC who were treated with cisplatin-based regimens.

		Clinical response(n = 100)			
		Response		Non-response	
	No	CR	PR	SD	PD
**CCND1 expression**					
Low expression	48	9	32	6	1
High expression	52	0	22	19	11
**Total**	100	9	54	25	12
* * ***P***		<0.001			

Note: Low expression: IOD score of CCND1 protein expression <31388.459;

High expression: IOD score of CCND1 protein expression ≥31388.459.

Through the analysis of CCND1 expression and survival in neoadjuvant group, we found that the DFS rates were significantly difference between low and high CCND1 expression subgroups, which were 63% and 6% (p<0.001, Fig. 2C). A similar result was observed with the OS rates for patients with low and high CCND1 expressions in neoadjuvant group, which were 73% and 8% (p<0.001, Fig. 2D). Similarly, in non-neoadjuvant group, the DFS rates for patients with low and high CCND1 expressions were 51% and 27% (p = 0.010, Fig. 2E), and the OS rates with low and high CCND1 expressions were 52% and 32% (p = 0.018, Fig. 2F).

### CCND1 expression is an independent risk factor

In the univariate analysis, CCND1 expression (p<0.001), lymph node status (p<0.001) and histologic signs of severity (p = 0.001) were associated with OS. The interaction between CCND1 expression and treatment was significantly associated with OS (p<0.001), suggesting a benefit from chemotherapy in neoadjuvant group patients with low CCND1 expressions, but a hazard from chemotherapy with high CCND1 expression when compared to non-neoadjuvant group. In the multivariate analysis that included the four factors (CCND1 expression, lymph node status, histologic signs of severity and the interaction of CCND1 expression × treatment), CCND1 expression (p<0.001), histologic signs of severity (p = 0.019) and lymph node status (p = 0.034) were found to be independent prognosis factors (Table 4).

**Table 4:** Cox proportional hazards regression models in estimating overall survival.

Variable	Hazard ratio	95% Confidence interval	*P*
**Univariate analysis**			
Age	1.008	0.995–1.022	0.238
Clinical stage	1.085	0.760–1.550	0.654
Pathologic grade	1.274	0.936–1.734	0.124
Smoking history	1.24	0.871–1.765	0.233
Alcohol history	1.279	0.886–1.845	0.189
Site (cat)*			0.337
Gender	0.685	0.451–1.041	0.077
Lymph node status	1.328	1.135–1.553	<0.001
Histologic signs of severity	1.845	1.277–2.664	0.001
CCND1 expression	3.223	2.216–4.689	<0.001
Treatment	0.948	0.671–1.339	0.762
CCND1 expression× treatment†	1.324	1.141–1.538	<0.001
**Multivariate survival analys**			
Lymph node status	1.199	1.014–1.419	0.034
Histologic signs of severity	1.58	1.078–2.317	0.019
CCND1 expression	3.638	2.194–6.033	<0.001
CCND1 expression× treatment†	0.906	0.738–1.111	0.341

Note: Site (cat).

*: categorical co-variable.

† : Interaction.

### CCND1 expression as a biomarker to predict who may benefit from neoadjuvant chemotherapy treatment

As the above analysis showed, CCND1 expression could predict prognosis in the two groups. When compared the OS in the two groups with low CCND1 expressions, we found that the patients from neoadjuvant group would receive more survival benefits than those from non-neoadjuvant group (p = 0.016, Fig. 2G). However, the patients from neoadjuvant group exhibited a more inferior OS than those from non-neoadjuvant group in two groups with high CCND1 protein expressions (p = 0.032, Fig. 2H). The results strongly indicate that the patients with low CCND1 expressions from neoadjuvant group may receive more of a survival benefit.

## Discussion

With regard to HNSCC, standard treatment consists of surgery followed by radiation therapy in high-risk patients. Neoadjuvant chemotherapy, including many regimens, has been investigated in head and neck cancer; however, the results remain inconclusive, if not negative [6]. A combination of cisplatin and fluorouracil has been shown to provide high response rates in untreated patients. Although many publications have reviewed the use of this regimen in advanced head and neck cancer, marginal tumoricidal activity at distant sites has been observed [5], [7], [24]. These results suggest that the regimens used were not potent enough to exhibit a therapeutic effect. In a previous clinical trial, we had found that cisplatin, teniposide or vindesine, and pingyangmycin were several of the most potent treatments for the patients with HNSCC as determined by a modified MTT chemosensitivity assay [25]. Thus, this cisplatin-based regimen has been frequently used in China over the past ten years. In this study, the total response rate reached 63% considering both T and N sites, while most of the patients received just one cycle of neoadjuvant chemotherapy.

Although many randomized trials have failed to show a survival advantage with the use of neoadjuvant chemotherapy, patients who achieved a clinical response had a more favorable prognosis [11], [26], [27], [28], [29]. In this study, we also found that the indiscriminate administration of neoadjuvant chemotherapy to patients did not pose a survival benefit but could present a hazard for survival.

The inability to choose the patients who would most benefit from chemotherapy was the primary cause of treatment failure. In this study, we found that CCND1 was an effective biomarker to predict the clinical response and prognosis of patients with HNSCC. A low CCND1 expression was closely correlated with chemotherapy response and favorable prognosis, whereas a high CCND1 expression may predict chemotherapy failure. The results are consistent with our previous studies showing that high CCND1 expression correlated with cisplatin resistance in oral cancer cells, *in vitro and in vivo* [21], [22]. Regardless of treatment with neoadjuvant chemotherapy or not, the patients with low CCND1 expression exhibited a better long-survival in comparison to those with high CCND1 expression. The results have shown that CCND1 expression correlated with prognosis is similar to previous reports [18], [19], [20], [21], [22].

It is interesting that patients with low CCND1 expression in the neoadjuvant group had a significantly better OS than those in the non-neoadjuvant group (73% *vs* 52%, p = 0.016); however, patients with high CCND1 expression in the neoadjuvant group had a worse OS in comparison to those in non-neoadjuvant group (8% *vs* 32%, p = 0.032). Furthermore, CCND1 expression and treatment had a significantly strong interaction in terms of prognosis. These results show that patients with low CCND1 expression have to receive the treatment with cisplatin-based neoadjuvant chemotherapy and surgery, whereas patients with high CCND1 expression should receive surgery-based treatment as early as possible rather than neoadjuvant chemotherapy plus surgery. According to multivariate analysis, we also found that CCND1 level was an independent prognosis factor in patients with HNSCC.

This study was retrospective and was restricted to patient subsets with samples; thus, all of the results are considered to be exploratory. In the present study, we chose the IHC method to evaluate cyclin D1 protein expression in HNSCC primarily because of the unavailability of fresh biopsy tissues. Although this method is a semiquantitative technique, IHC analysis is the most commonly used, simplest, and cheapest protocol in clinical work [30]. Moreover, it is believed that cyclin D1 protein overexpression may occur via other mechanisms besides gene amplification, and the measurement of protein levels would be more informative than cyclin D1 DNA copies [19], [31], [32], [33]. According to the obtained results, low cyclin D1 expression at pretreatment forecasts a better clinical response and an improved DFS and OS in neoadjuvant chemotherapy patients.

In this paper, a portion of sections were added P-CK stain to test the accuracy of CCND1 scoring area and to rule out the non-cancerous cell stain. The results showed that the determined area and cells which were chosen by researcher and pathologists were specific and typical. The status of CCND1 expression (low or high expression) was not changed after these sections reappraised according to the area and cells of the positive P-CK stain. Therefore, we have believed that the CCND1 stain alone combined with irregular AOI function of IPP 6.0 should be a reliable method of IHC scoring. And we have assumed that the method of score by IPP software is more objectivity than microscope count by observer. So the method may be the scoring trend of dyeing experiment such in future.

In conclusion, our study indicates a key role of CCND1 in determining chemotherapy response and prognosis. We can select the patients with HNSCC who have the greatest chance of benefiting from neoadjuvant chemotherapy by CCND1 expression. Indeed CCND1 expression may serve as a predictive biomarker in selecting patients undergo future neoadjuvant chemotherapeutic clinical trials.
